# Lessons from HeLa Cells: The Ethics and Policy of Biospecimens

**DOI:** 10.1146/annurev-genom-083115-022536

**Published:** 2016-03-03

**Authors:** Laura M. Beskow

**Affiliations:** 1 Program for Empirical Bioethics, Duke Clinical Research Institute, Duke University School of Medicine, Durham, North Carolina 27705; 2 Department of Medicine, Duke University School of Medicine, Durham, North Carolina 27710

**Keywords:** informed consent, biological specimen banks, public opinion, policy making, confidentiality, trust

## Abstract

Human biospecimens have played a crucial role in scientific and medical advances. Although the ethical and policy issues associated with biospecimen research have long been the subject of scholarly debate, the story of attention of a much broader audience. The story has been a catalyst for policy change, including major regulatory changes proposed in the United States surrounding informed consent. These proposals are premised in part on public opinion data, necessitating a closer look at what such data tell us. The development of biospecimen policy should be informed by many considerations—one of which is public input, robustly gathered, on acceptable approaches that optimize shared interests, including access for all to the benefits of research. There is a need for consent approaches that are guided by realistic aspirations and a balanced view of autonomy within an expanded ethical framework.

## INTRODUCTION

Human biospecimens have played a crucial role in scientific and medical advances, and their continued widespread availability for research will be vital to realizing the goals of precision medicine (13). Discoveries from biospecimen research have led to new understandings of human biology and targeted approaches to detecting and treating health conditions, as well as reducing the risk of future disease. In oncology research, for example, biospecimen use has increased dramatically in recent decades (67), helping illuminate molecular mechanisms that drive cancer and generating knowledge that, in some instances, has profound implications for risk assessment, diagnostic categorization, and novel therapeutic strategies (26).

The collection, storage, and research use of biospecimens and data, however, raise deep questions about informed consent, oversight, large-scale data sharing, privacy and confidentiality, commercialization, access to research results, and the ability to withdraw (94). The success of this enterprise critically depends on addressing such concerns in ways that are acceptable to patients and the public, and on building and maintaining support, trust, and transparency.

Although the ethical and policy issues associated with biospecimen research have long been the subject of scholarly analysis and debate (48, 118, 124), the publication of Rebecca Skloot’s bestselling book *The Immortal Life of Henrietta Lacks* (130) captured the attention of a much broader audience. The book is a popular teaching tool and book club favorite (138), and the book and its author have been the subject of numerous reviews, news stories, features, commentaries, profiles, and interviews (99).

The story of Henrietta Lacks, her family, and the creation of HeLa cells has been a catalyst for policy change (64), including major regulatory changes proposed in the United States for informed consent for biospecimen research. This review reflects on the role of the HeLa controversy and public opinion data more generally in the development of biospecimen research policy, and the need for informed consent approaches that are guided by realistic aspirations and a balanced view of autonomy within an expanded ethical framework.

## REFLECTIONS ON THE STORY OF HENRIETTA LACKS

The story behind the HeLa cell line is now widely known (129): Henrietta Lacks, a 30-year-old African American woman with five children, was diagnosed with an unusually aggressive form of cervical cancer at Johns Hopkins Hospital in 1951. Tissue samples were taken during her diagnosis and treatment, and portions were passed along to a researcher without her knowledge or permission, as was common practice at the time. Researchers had long endeavored without success to grow human cells outside the body, and it soon became clear that Henrietta’s cancer cells—labeled “HeLa” based on the initial letters of her first and last names—were capable of surviving and dividing in culture indefinitely. The cancer quickly took Henrietta’s life, but HeLa cells remain viable today and have been used in laboratories around the world for a vast array of biomedical research. Although the original researchers gave the cells away to anyone who asked, the cell line and downstream discoveries became extremely lucrative—while the Lacks family received no financial benefits and continued to live in poverty with limited access to health care.

The 2010 publication of *The Immortal Life of Henrietta Lacks* (130) garnered widespread attention. Nisbet & Fahy (99) found that, in the popular press, informed consent dominated discussion of the book. The welfare of the vulnerable and compensation were also prominent themes. Scientific progress, patient control, and accountability were discussed to a lesser degree, and privacy, public education, and advocacy even less so. Discussion in professional literature comprised a similar array of themes, including marked emphasis on informed consent (20, 22,33,37, 49, 79, 123, 135, 147), as well as commercialization and compensation (123, 141); privacy and confidentiality (20, 33, 49); race, poverty, and health disparities (42, 123); familial implications of genetic information (14, 22,64, 96, 135); ownership of biospecimens (22, 123); and trust in biomedical research (135).

Despite heightened scrutiny of these issues, a team of researchers posted the whole genome sequence of one strain of HeLa cells online in 2013. Doing so broke no laws or rules; large-scale sharing of genomic data sets is required by many funding sources (107) and journals to promote replication of findings and further research. However, because these data provided some probabilistic information about Henrietta Lacks and her descendants, now known to millions by name, criticisms concerning privacy and informed consent intensified (131). In response, the researchers removed the sequence from the public domain, and the director of the National Institutes of Health (NIH) met with the Lacks family (49). An agreement was reached by which NIH-funded researchers who sequence HeLa cell lines are expected to deposit the data in a controlled-access database; applications to study these data are reviewed by a committee that includes members of the Lacks family (64).

Although Henrietta Lacks’s story is compelling and has prompted much-needed public discussion, it is an extraordinary case in many respects. Rarely do biospecimens obtained from one individual prove particularly valuable (15, 74,119, 134, 141, 145). More typically, scientific discovery and translation require the study of biospecimens and data from hundreds—if not hundreds of thousands—of people (112), with and without the condition of interest, over many years. Furthermore, the original source of HeLa cells is decidedly famous; usually, identifying the source of a genetic sample from which identifiers have been removed would require intent and technical wherewithal, as well as motivation and a means by which to exploit this information (117).

Caution is warranted in drawing lessons for biospecimen policy from any exceptional case. However, the HeLa story aptly illustrates enduring and escalating questions about research use of human biospecimens. Some of these issues have been analyzed based on legal concepts of property rights, ownership, and invention (38). This review focuses primarily on ethics and policy questions around informed consent, including current and proposed regulations and future directions.

## INFORMED CONSENT FOR BIOSPECIMEN RESEARCH

Informed consent describes a process for enabling individuals to make voluntary decisions about participating in research with an understanding of the purpose, procedures, risks, benefits, and alternatives. Informed consentis premised on well-established ethical principles, including respect for persons, beneficence, and justice (98). Following from these principles, key aspects of informed consent include the provision of information about the research that a reasonable person would want to know, in a manner and language understandable to the person, and under conditions that are free from coercion or undue influence.

Several approaches to informed consent for the research use of biospecimens have been suggested (Table 1). Actual practice varies, as collections of biospecimens (or biobanks) are highly heterogeneous in terms of tissue type, procurement situation, and geographic, social, and historical context (56, 58,60, 126, 146). Furthermore, regulations and guidelines concerning informed consent are not necessarily specific to biospecimen research, nor are they harmonized (40, 59). As illustrated by current US regulations, the result has sometimes been ambiguity, inadvertent constraints on research access, and research proceeding without consent (40, 47, 69).

## Current US Regulations

In the United States, federal regulations (known as the Common Rule) were developed in response to revelations of extreme research abuses of vulnerable populations; these regulations were designed primarily to protect human beings from physical risks involved in experimental research. They set forth provisions for informed consent and oversight by an institutional review board (IRB) that, with limited exceptions, must be met in federally funded research.

In terms of application to biospecimen research, the Common Rule defines a human subject as a living individual about whom an investigator obtains (*a*) data through intervention or interaction with the individual or (*b*) identifiable private information. Thus, when an investigator interacts with a person to collect biospecimens specifically for research (e.g., for a particular study or to build a biobank), informed consent and IRB oversight are required. However, when an investigator uses only biospecimens that have already been collected for another purpose (e.g., for a clinical purpose, for an earlier study, or to build a biobank), no intervention or interaction with a person is involved. Furthermore, a fundamental strategy to protect confidentiality is to remove direct identifiers and replace them with a code, and take additional steps to ensure that researchers have no access to identifying information. For example, material transfer and data use agreements can prohibit access to the key that links the code to identifiers (the code cannot be derived from information about the person; thus, Henrietta Lacks’s cells today would not be labeled HeLa), as well as any attempt to reidentify sample sources.

Under these conditions—when there is no intervention or interaction with the individuals who were the sources of the samples, and researchers cannot readily ascertain their identities—research can be determined not to involve human subjects (143), and thus informed consent is not required. There are other provisions that allow research using existing biospecimens to be classified as exempt from the regulations (e.g., if researchers do not record identifying information), in which case consent would not be required, as well as provisions for waiving the requirement to obtain consent (when research qualifies as nonexempt research involving human subjects but certain criteria are met). All of these situations require some level of IRB involvement, as investigators cannot make these determinations themselves. Rather, they must submit at least basic information to an IRB, at which point the IRB can assess the adequacy of confidentiality protections, and could choose to consider the scope of the original consent (if any) and whether reconsent is required.

Thus, if Henrietta Lacks were a patient in the United States today, biospecimens collected solely for her clinical care would not require her consent for use in research. Any part of such specimens remaining after all the analyses needed for her care were completed might be stored for generic teaching, quality assurance, and research purposes, as briefly disclosed in a general consent-to-treat form. Researchers seeking to study stored clinical specimens could do so without her consent if an IRB determines either that the proposed research does not involve human subjects (based primarily on their having no access to identifiers) or that it meets the criteria for exemption from the regulations or waiver of the requirement to obtain consent. An IRB-approved research protocol and informed consent (unless waived) would be required when researchers prospectively intend to use clinical specimens for a specific project, including plans for use of residual specimens as well as taking more tissue than is needed for clinical care (i.e., taking extra tissue for research purposes during a necessary clinical procedure). Collecting biospecimens from family members solely for research purposes would require an IRB-approved protocol and informed consent.

In summary, regulations originally intended to protect research participants from bodily harm have been interpreted and clarified through guidance and practice to apply to research on biospecimens, with identifiability as a pivotal factor. However, this rapidly evolving research arena has been accompanied by equally rapid confirmation that genomic data can never be truly anonymized. A steady stream of provocative studies (51, 62, 68, 122) has demonstrated that it is possible to discover the identities of individuals whose genomic data had otherwise been considered deidentified. These developments come at a time when public concern about biospecimen research and informed consent has already been stoked not only by the story of Henrietta Lacks, but also by lawsuits over the research use of newborn screening samples (18, 30) and biospecimens from indigenous populations (28, 95).

## Proposed US Regulations

In September 2015, the federal government published a notice of proposed rulemaking (NPRM) to overhaul the Common Rule (102). Citing the changing research landscape, including the volume and diversity of studies, analytic sophistication, and growing use and global sharing of massive electronic data sets, the stated goals of the NPRM are to increase human subjects’ability and opportunity to make informed decisions, reduce potential for harm and increase justice by increasing the uniformity of protections, and facilitate promising research. Among the most significant changes are those proposed for biospecimen research:

■ The definition of a human subject would be modified to include living individuals about whom an investigator “obtains, uses, studies or analyzes biospecimens” (102, p. 54004), regardless of identifiability.
■ With few exceptions, consent would be required for research use of all biospecimens— regardless of whether they were originally collected for research, clinical, or other purposes and whether they are deidentified (notably, consent would not be required for secondary research use of nonidentified private information, such as medical records).
■ Consent would not be needed for each specific study, but rather could be obtained through broad consent for future unspecified research.
■ The government would develop a broad consent template covering the storage of biospecimens and data for secondary research, as well as the use of stored materials for specific studies.
■ In addition to basic elements of consent, additional required elements would cover commercial use and profit from study of biospecimens, return of individual research results, optional recontact, and widespread sharing, among others.
■ Secondary use of biospecimens to establish a biobank would be exempt, subject only to limited IRB review to ensure that initial broad consent has been obtained and specified privacy and security safeguards are in place.
■ Research using materials that have been stored for secondary use would be exempt from the regulations and required only to have safeguards in place (with no IRB review). Investigators could use a to-be-created decision tool to make the exempt determination themselves.

As one commentator noted, the NPRM “manifests a near-obsession with the rules governing biospecimens, resulting in what some critics call biospecimen exceptionalism” (34, p. 2299). Although the NPRM does not mention Henrietta Lacks, her influence is clear in a publication about the proposed changes authored by leaders of these reforms (65), which stated that “the controversy that followed the 2013 publication of the genome sequence of the HeLa cell line…underscores the need for greater involvement of and respect for research participants” (p. 2293) and that “just as the Lacks family’s experience helped clarify how research needed to change, the perspectives of researchers, the public, and patients should be heard as these reforms are finalized” (p. 2296).

The proposed changes have been the subject of intense debate. With regard to the collection of biospecimens specifically for research, the ethical acceptability of broad consent for future unspecified use has long been discussed (21, 52, 54, 61, 75, 109), but surveys suggest that it is now common in the United States (57) and possibly other countries (40, 109). Routinely obtaining consent for the research use of residual clinical specimens, however, would be a substantial departure from current practice (3). Although some support the NPRM’s clarification and specification of procedures for biospecimen research (34), others say the consequences will be missed opportunities and lost lives caused by delays in important research (50). One commentator predicted that obtaining consent from patients for secondary use of clinical specimens would “become perfunctory—another form to sign during admission—and fall far short of our goal of respect for persons; add a large, complex, and expensive burden to institutions; and create new barriers to the conduct of research” (17).

The NPRM justifies these changes based in no small part on appeals to public opinion, stating that “continuing to allow secondary research with biospecimens collected without consent for research places the publicly-funded research enterprise in an increasingly untenable position because it is not consistent with the majority of the public’s wishes, which reflect legitimate autonomy interests” (p. 53944) and that “most importantly, people want to be asked for their permission. A growing body of survey data show that many prospective participants want to be asked for their consent before their biospecimens are used in research” (p. 53938). Given this reliance on public opinion, what do such data suggest regarding consent for biospecimen research?

## PUBLIC OPINION ON CONSENT FOR BIOSPECIMEN RESEARCH

In support of proposed regulatory changes, the NPRM cited four articles reporting public opinions about biospecimen research (77, 128, 140, 144). But as summarized below, these publications do not necessarily suggest consensus.

First, Kaufman et al. (77) reported the results of a national online survey conducted among US adults in 2007–2008. The purpose was to assess the conditions under which the general public might participate in a biobank established and operated by the NIH and other federal health agencies. Survey participants (*n* = 4,659) received a short description of the hypothetical project, which involved a physical exam, collection and genetic analysis of blood samples, and periodic surveys concerning general health, lifestyle, and environmental exposures. These materials would be stored in coded form at the NIH, and researchers would apply to study them to learn more about how genes, environment, and lifestyle contribute to health and disease.

Although 90% of respondents said they would be somewhat or very concerned about privacy, 60% said they definitely or probably would be willing to participate. With regard to consent, 48% said they would prefer to give permission once, at the beginning of the study, for all research approved by an oversight committee. Slightly fewer (42%) said they would prefer to be asked for permission for each research project separately, and only 10% preferred to select categories of research for which they would let their materials be used.

In a more recent publication (not cited in the NPRM), Kaufman and colleagues (110) winnowed the full data set from the 2007–2008 survey by various criteria and analyzed a subset (*n* = 3,347) in which 51% preferred broad consent and 49% preferred study-specific consent. In this subset, older, male, and white non-Hispanic participants were more likely to prefer broad consent. However, these differences were explained by logistic regression models that included responses to questions regarding beliefs about the study: Broad consent was preferred by those who agreed that the study could lead to improved treatments, cures, and lives saved; that participating would make them feel like they were contributing to society; that participating would be easy; and that they would feel bothered by researchers asking permission for each use. Study-specific consent was preferred by those who expressed concern about researchers having their samples and information and about the possibility that information collected could be used against them, and who said that being asked permission for each use would make them feel respected and involved.

A second study cited in the NPRM was conducted by Vermeulen et al. (144) at one cancer institute in the Netherlands in 2007–2008. The purpose was to ascertain preferred consent procedures for research use of residual clinical specimens among patients who had undergone primary surgery for breast, prostate, or colorectal cancer. Participants (*n* = 133) were randomized to either a onetime consent procedure or an “opt-out plus” procedure. Both groups received brief verbal information from a health professional, as well as a four-page leaflet to read at home. Those in the onetime consent group were told, “Please read this leaflet at home and send back the consent form within 1 month in the stamped return envelope indicating whether you consent to the use of your tissue for future medical research” (144, p. 1506). Those in the opt-out plus group were told, “Please read this leaflet at home. You may want to object to research with your tissue and you may do so by sending back the form in the stamped return envelope” (144, p. 1506). Notably, this intervention took place during a postsurgical visit to avoid burdening patients during early diagnosis and treatment. A control group (*n* = 131) received a general hospital leaflet that informed patients about, among other things, the possible use of stored tissue for research. This short passage included the statement “Patients are informed that they can opt-out if they wish by informing their physician, who will then make a note in the medical charts” (144, p. 1507).

A follow-up questionnaire (completed by approximately 90% of each group) indicated the following:

■ Among the respondents, 82% of those in the onetime consent group and 65% of those in the opt-out plus group felt well informed; by contrast, 73% of those in the control group had not seen or did not remember having read the information about tissue research.
■ Among all respondents, 43% preferred opt-out plus, 34% preferred onetime consent, 16% preferred the standard opt-out procedure, and 8% said that no information at all was needed.
■ Respondents who were younger, female, and more highly educated and considered themselves to be the owners of their tissue were more likely to prefer onetime consent and opt-out plus over standard opt-out. In all subgroups, opt-out plus was preferred more often than other approaches.

A third study cited in the NPRM was conducted by Simon et al. (128) in 2010. Seven focus groups (*n* = 48) and a telephone survey (*n* = 751) were conducted among English-speaking adults in the catchment area for a biobank being developed at the University of Iowa. The study population was relatively homogeneous; for example, survey respondents were predominantly white and female and had high levels of income and education. Focus group input was based on a description of biobanks as being typically managed by a medical center and involving samples that could be either left over from a clinical procedure or collected for research. Survey participants responded to a scenario specifically about a proposed biobank at the University of Iowa that would store residual clinical samples that would otherwise be discarded. In response:

■ A majority (63% of focus group participants and 67% of survey participants) preferred an opt-in approach to initial consent. Common reasons included that opt-in provided the opportunity to make a more positive, active, and informed choice and would receive greater public acceptance as fitting with traditional notions of consent. Opt-out was preferred by 25% and 18% of focus group and survey participants, respectively. “No consent” was discussed but not polled in the focus groups, and was the preferred approach for 5% of survey respondents.
■ In terms of scope, broad consent was the most commonly preferred (54% of focus group participants and 41% of survey participants), for reasons such as greater ease and flexibility for research (and thus a boost to research output) and recognized uncertainties regarding future research. Study-specific consent was preferred by 21% and 29% of focus group and survey participants, respectively, and categorical consent was preferred by 21% and 25%, respectively.

The authors noted that, theoretically, the proportions that preferred study-specific and categorical consent could be combined on the grounds that both models promote control and choice over future use. If so, a general control/choice model could be seen as preferred (by 42% and 54% of focus group and survey participants, respectively) over broad consent. Even so, they concluded, “Many individuals may want to make an active and informed choice at the point of being approached for biobank participation but are prepared to consent broadly to future research use and to forego additional choices as a result” (128, p. 821).

The final article cited in the NPRM was a commentary by Trinidad et al. (140) that informally referred to a few findings from research at Group Health, a nonprofit health care system in Seattle, Washington. This research included efforts to contact individuals who had consented to a cohort study of aging and dementia [called Adult Changes in Thought (ACT)] in order to obtain additional consent for widespread data sharing, a survey of ACT participants who did provide this reconsent, and focus groups with ACT participants and Group Health patients (139) about large-scale genomic research. Themes mentioned in the commentary included trust in Group Health and ACT investigators, altruistic motivations, and little concern about privacy, confidentiality, or discrimination. The authors concluded that many participants view themselves as having an ongoing stake in research to which they have contributed, and “want to be asked (or at least kept informed) about changes in research” (140, p. 288).

In summary, the publications the NPRM cited in support of its contention that “a growing body of survey data show that many prospective participants want to be asked for their consent before their biospecimens are used in research” (p. 53938) actually provide a highly complex picture that does not necessarily fit the proposed regulations. These articles represent perspectives from decidedly different “publics” who were asked about different kinds of biobanks and presented with different options from which to indicate their preference. In general, the option of “no consent” was seldom explored (and was seldom applicable to the scenarios involved in these studies). Broad consent was preferred in one study, with study-specific consent a close second, whereas in another study, an opt-out procedure involving verbal notification was clearly preferred over broad consent. Few participants in these studies selected categorical consent as their preferred model—but if these participants are combined with those who wanted study-specific consent, one could deduce a desire for more choice and control.

Beyond these particular studies, lay attitudes toward biobanking have been the focus of considerable empirical research, and the same challenging picture emerges. Since 2010, this literature has been the subject of at least seven published reviews (Table 2). These reviews encompass a wide array of studies that differ substantially in context, purpose, design, samples, results, and conclusions. Although the reviews themselves all centered on studies of public, patient, and/or participant perspectives, they varied widely with regard to stated research question(s), methodology, and inclusion/exclusion criteria. Some of the reviews rated the quality of the evidence (24, 43), but their synthesized findings are limited by nontrivial limitations in the underlying studies. As stated by Gottweis et al. (45), “most of these studies yield interesting data, but currently we are confronted with a heterogeneous patchwork of insights rather than a coherent picture of evidence” (p. 434).

## ELICITING AND USING PUBLIC OPINION

### What We Ask and How We Ask It

Input from the public, patients, and research participants is essential for informing the development of sound research policy. Robust mixed-methods approaches, combining both quantitative and qualitative techniques, can help to both elucidate and explain patterns of attitudes, opinions, beliefs, and actions (45). Many if not most people have no preexisting knowledge or opinions about biobanking or the ethical and policy issues and controversies so familiar to “experts.” Thus, careful attention is needed to what we ask and how we ask it.

With regard to what we ask, a substantial portion of empirical research to date has asked about preferences. What individuals might prefer, however, is not the same as what they might find acceptable, once they are aware of the risks, benefits, costs, and trade-offs at stake for the array of interests they would like to see advanced. Two examples readily demonstrate the difference between asking about preferences and asking about acceptability:

■ In the study by Simon et al. (128) summarized above, participants in the telephone survey were read a description of each consent model and asked whether they supported or opposed it. Participants were then asked which model they most preferred. The publication focused primarily on the preference results, but the one reported data point on support is telling: An opt-out approach to initial consent was the preferred choice for only 18%, but it was supported by 78%. The reasons for finding opt-out acceptable (even if not preferred) were that it would allow for at least some measure of knowledge, choice, and control and would contribute to increased biobank accrual, cost less time and money, and spur scientific progress and discovery.
■ As noted above, in the study by Kaufman et al. (77), 48% of respondents preferred broad consent and 42% preferred study-specific consent. Some members of the same research team recently conducted a very similar online survey of US adults (36) in which participants were randomly assigned to one of two scenarios in order to examine acceptance of broad consent (*n* = 1,528) versus study-specific consent (*n* = 1,533). Acceptance was measured by stated willingness to participate in a hypothetical biobank study. Willingness to participate was substantially higher than the proportions suggested by the earlier preference data and did not differ between groups: 76% of those who received the description of broad consent and 74% of those who received the description of study-specific consent said they would participate.

In contrast to clinical care, the goal of research is not to optimize individual preferences, but rather to generate socially valuable scientific knowledge (72). Therefore, to help inform research policy, studies of public opinion should focus on, or at a minimum include, questions exploring the acceptability of various approaches to ethical challenges (see sidebar, Asking Patients About Acceptable Policy Options).

In terms of how we ask, attitudes and opinions are often explored using hypothetical scenarios. This technique is frequently criticized with regard to how accurately the responses generated predict real-life behavior. For example, Johnsson et al. (73) compared hypothetical to factual willingness to participate in biobank research in studies matched by country and time frame. Among 22 pairwise comparisons, 12 suggested that factual willingness was greater than hypothetical, 6 indicated the reverse, and 4 were inconclusive. The authors concluded that motivational factors, such as altruism, trust, and sense of duty, may be less influential in hypothetical contexts, and thus the value of such scenarios in predicting factual willingness to participate may be limited.

The use of hypothetical scenarios—like all methodologies—has disadvantages, but it also has important advantages as a practical, flexible, and efficient way to study complex ethical concerns (142). In the face of emerging issues, hypothetical scenarios can be used to anticipate public reactions, manipulate key contextual variables, and contribute to the development of evidence-based interventions to preempt adverse outcomes (108). As with any research technique, rigor is essential. Scenarios must be empirically developed to be valid and reliable, and with characters, social context, and situations presented in a way that is authentic, relevant, and meaningful to participants (142). Attention to features such as verbal immediacy, temporal proximity, level of detail, format of response options, and cognitive demand may help improve predictive accuracy (108). Similar to the difference between asking about preference and asking about acceptability, constructing hypotheticals in second-person as opposed to third-person language matters, as does eliciting “should” as opposed to “would” responses (66).

### The Role of Public Opinion in Policy Development

Even in a world of ideally designed and conducted studies, the role of public opinion in developing research policy on ethically challenging topics is a fundamental question. There is no doubt that understanding public perspectives on these issues is vital, and as illustrated by the destruction of millions of newborn bloodspots, failure to take it into account can have harsh consequences (69). There are, however, several reasons why public opinion alone should not dictate policy: (*a*) Data on public perspectives are not definitive (21, 92, 120); (*b*) attitudes and opinions may be expressions of intense moral reflection or the product of misunderstanding, bias, selfishness, self-deception, and other problematic factors (116, 120); and (*c*) normative judgments must be grounded in, or at least reconciled with, foundational principles (21, 116, 120). Public input is one factor among many that should inform policy, and this input can be invaluable for assessing public understanding and areas of concern, identifying previously unrecognized ethical issues, describing facts relevant to normative arguments, avoiding solutions likely to be rejected by many, and learning from people’s lived experiences (32, 86).

### WHERE TO FROM HERE?

The story of Henrietta Lacks and HeLa cells generated tremendous public attention—particularly around informed consent for biospecimen research—and has demonstrably influenced policy discussions in this arena. These have occurred in the context of rapid scientific and technological innovation and the inescapable recognition that complete deidentification of biospecimens and data is illusory. As a solution, proposed regulatory changes look prominently to routine consent for research use of biospecimens, regardless of whether they were originally collected for research or clinical purposes. These proposals are premised in part on public opinion data, although a closer look at such data reveals a complicated mosaic of perceptions and preferences. What lessons can we draw from all this about ethics and policy for biospecimen research?

### Expectations of Informed Consent

First and foremost, informed consent cannot bear the weight it is being asked to shoulder (16, 81). There is a chasm between the theoretical ideals of informed consent and what it accomplishes in actual practice (46, 53, 55). Empirical research has amply shown that consent forms are too long and written at too high a grade level (1, 4,19,29, 76, 84, 85, 105, 106, 125) and, not surprisingly, many participants do not understand the information disclosed (4–6,70,71, 83, 87, 97), including biobank participants (93,104,114). This situation has led commentators to note that consent forms are “growing in length and complexity, becoming ever more intimidating, and perhaps inhibiting rather than enhancing participants’ understanding. Participants may not even read them, much less understand them” (35, p. 10).

Numerous interventions have been tried to improve informed consent processes and participant understanding, including enhanced forms, multimedia approaches, test/feedback procedures, and extended discussion. These attempts have met with minimal success; across four systematic reviews (25, 39, 100, 136), the only intervention consistently shown to be effective was one-on-one discussion with a person knowledgeable about the study. However, all four reviews found the available evidence notably limited, with the studies often suffering from lack of power, nonrandomized designs, poor generalizability, questionable methods for measuring comprehension outcomes, and/or too narrow a focus on a single aspect of the consent process.

At Duke University’s Program for Empirical Bioethics, we have conducted a body of research on informed consent for biobanking (8, 11, 12), including development of a simplified consent form (10) and a consensus-based definition of what constitutes adequate comprehension (9). We hope the latter, which resulted from a systematic explication of the minimum knowledge individuals must demonstrate to provide valid consent, might prove useful for improving consent forms and processes and as an absolute metric for assessing the effectiveness of other interventions to improve comprehension.

However, even if the gap between theory and practice were narrowed, informed consent is not a panacea. There is little question that Henrietta Lacks and her family should have been given more information and asked for their permission for researchers to take and use her tissue—but we should be wary of resting easy that consent would have been the whole answer (123). Particularly when patients are facing dire situations, we cannot simply assume that an elicited choice will be actively and freely made based on careful consideration and adequate understanding of the information (27).

As another example of the limits of even idealized informed consent, merely disclosing that confidentiality cannot be guaranteed does not absolve researchers and policy makers of the responsibility to strengthen protections. Solutions to the identifiability problem that better support the interests of all stakeholders would focus not only on disclosing risks to prospective participants, but also on minimizing potential harms by ensuring the scientific merit of proposed studies (116) and prohibiting misuse of information (33, 121).

Finally, too much weight on consent elevates autonomy—a word referenced 43 times in the NPRM—as the guiding ethical principle at a high cost to other principles and values (17). As Taylor (137) eloquently asserted, no ethical principle has transformed biomedicine as powerfully as autonomy—and yet, when “ethics is reduced to autonomy, autonomy is reduced to naked choice, and a self-commodifying model of choice is substituted for richer visions of human nature and interdependence” (p. 32).

### New Ethical Frameworks

Given the immense changes in the landscape of biomedical research, one response would be a major initiative to update the ethical framework that provides the intellectual foundation for developing and evaluating policy strategies. For example, with regard to genetic databases, Chadwick & Berg (23) described a duty to facilitate research progress and provide knowledge that could be crucial to the health of others (particularly when risks and burdens are minimal) and for the benefits of research to be shared widely. Knoppers & Chadwick (80) later expanded on emerging trends in human genetic research, away from individualism and autonomy as paramount and toward more participatory ideals (Table 3). Critical to these trends is the recognition that any increased expectation that people will participate in research must be balanced with an imperative that the benefits of research be accessible to all.

More generally, a modernized framework for research ethics could, for example, help develop a coherent view of whether people who are the sources of biospecimens are research participants or donors, and whether their specimens are a gift or a contribution—distinctions that lead to different conceptualizations of relationships among people, their biospecimens, and researchers, as well as different views of appropriate policy solutions (45, 69). Such a framework could also lead to more deliberate ethical grounding for solutions beyond traditional, study-specific informed consent. Interestingly, the article by Trinidad et al. (140), which was cited in the NPRM in support of the expanded use of consent, emphasized other innovations, such as better communication about research being done with biospecimens and data (7), transparent and accountable oversight processes (41, 148), and opportunities for community engagement and input on stewardship of data (63, 103).

### Alternatives to Traditional Consent

In the meantime, with regard to informed consent per se, a recent report from an expert workshop convened by the NIH Clinical Center’s Department of Bioethics articulated both the benefits and costs involved in asking people for consent to use their biospecimens in research (47) (Table 4). Weighing these considerations, as well as public opinion data, most workshop participants endorsed the use of broad consent in both clinical and research settings when it is coupled with oversight and, when feasible, ongoing provision of information to participants. Their restriction regarding oversight is key. Broad consent is commonly conceptualized as consent to governance (82, 91), and studies of public opinion about broad consent are predicated on descriptions that involve entrusting decisions about specific studies to an ethics review board or other oversight body. In proposing the standardized use of broad consent, a significant shortcoming of the NPRM is that it removes IRB review of secondary uses of biospecimens and does not contemplate any other oversight mechanisms independent of the researchers.

Moreover, active opt-out procedures for the research use of residual clinical specimens merit further consideration (44). In contrast to traditional opt-out, in which a brief mention of research use may be buried in a consent-to-treat form, actively notifying patients that their biospecimens may be used for research and alerting them to their right to opt out arguably affords the same benefits elucidated in Table 4, but at a lower “cost.” Many have urged that such an approach is in fact optimal for clinical specimens when certain conditions are met:

■ *Transparency*: Committed effort must be made to raise patient awareness that the default position is for residual tissue to be included in research (44). For instance, patients could be provided with a brief verbal explanation and directed to simple written materials [per the opt-out plus model described by Vermeulen et al. (144)], and their awareness could be reinforced through video spots on waiting room and patient room TVs, email and smartphone messaging, newsletters, brochures, posters, websites, and newspaper and radio stories (16).
■ *Sufficient information*: Patients need adequate information to make a choice (16)—our consensus-based definition of adequate comprehension for biobanking consent (9) may provide a useful starting point—and should have ready access to additional details if desired (44).
■ *Genuine opportunity to object*: Patients have an opportunity to make a meaningful choice when institutions make an honest attempt at transparency, actively provide sufficient information, and ensure easy means by which patients can register a decision to opt out (16, 44).

Finally, other nontraditional approaches to informed consent may be appropriate for certain populations and studies, including open (2, 90), dynamic (78, 127, 132), meta (111), and cascading (89) consent. However, despite their appeal, these models are also not a cure-all. Given the general limitations of informed consent, the creation of ever more elaborate processes raises the specter of what has been called “the tyranny of choice” (115), as well as legitimate questions about the claims for these models regarding increased autonomy, engagement, control, and reciprocity (133).

## CONCLUSION

Henrietta Lacks’s story rightly prompted far-reaching discussions about a range of ethical and policy issues in biospecimen research. Although the creation of HeLa cells occurred decades before our current system of human research protections, recent events surrounding the publication of HeLa sequence information underscore the pressing need for policy changes that are based on living ethical frameworks and are equal to the challenges presented by the revolution in genomic and other big-data science.

The development of biospecimen policy should be informed by many considerations, one of which is public input, robustly gathered, on acceptable approaches that optimize vital shared interests. The unique story of Henrietta Lacks and her family provides an opportunity to reflect on these interests, including the importance of recognizing and respecting every individual, building and maintaining the trust of patients and the public, facilitating research that holds the promise of contributing to the goal of alleviating suffering and improving human health, and meeting the moral obligation to ensure that the benefits of such research are available to all.

## Figures and Tables

**Table 1:** Models for obtaining permission for research use of biospecimens

Model	Description
No consent	Individuals are not approached for permission for research use of biospecimens.
General notification	Individuals are actively or passively alerted to the research use of biospecimens and offered an opportunity to opt out (where the default is that biospecimens will be used unless the individual refuses) or opt in (where the default is that biospecimens will not be used unless the individual agrees).
Blanket consent	Individuals are asked to consent to all future research with no limitations or conditions.
Broad consent	Individuals are asked to consent to the collection and storage of biospecimens for future unspecified research, which will occur under conditions defined at the time of consent (e.g., oversight or right to withdraw).
Categorical consent	Individuals are asked to consent to the collection and storage of biospecimens for future research use and are offered a checklist of options to stipulate by whom and in what ways they can be used.
Dynamic consent	Individuals are provided with an interactive, digital system that allows them to tailor, modify, and update consent choices as their circumstances change and in response to specific studies.
Study-specific consent	Individuals are contacted and asked for consent for each research use.

**Table 2:** Summary of review articles on lay attitudes toward biobanking

Review	Description	Main findings	Conclusion
Gottweis et al. 2011 (45)	Narrative review of 23 quantitative and qualitative studies of public perceptions of biobanks, 2003–2010 (international)	■ Overall, existing studies on public perception of biobanks tend to concentrate on certain regions. They differ with respect to methodological conceptualization and research design, are typically comparable only to a limited extent, and often present contradictory data. ■ Apart from local variations in biobank perception, people clearly differentiate between biobanks based on their type and purpose. ■ The link between knowledge about biobanks and preferred consent forms and readiness to participate in biobank studies is clearly an issue of great importance. ■ People want to know about the entity to which they are entrusting their data. ■ Most studies to date had suggested a generally low level of knowledge on biobanks and genetic research by different publics.	“We argue that the existing data originate in a relatively few regions, among them Northern Europe, the United Kingdom, and in certain U.S. states and are often based on survey research with small samples and short questionnaires. Combined usage of qualitative and quantitative methodology in studies is still rare though of great importance in order to investigate distributions of public opinion and also to be able to explain these patterns. Many important questions in the relationship between publics and biobanks are unexplored, or the existing data are inconsistent.” (p. 433)
Lipworth et al. 2011 (88)	Narrative review of 36 qualitative studies of lay perceptions of biobanking, 2002–2009 (international)	■ Any “sociology of biobanking” would need to be nuanced and draw on a variety of social theories in order to account for the donor population, the type of tissue being donated, and the context of the donation. ■ For the most part, people are not a priori against commercially funded research; few people feel a strong sense of ownership of their tissue; and where an expectation of reciprocity does emerge, it appears to be centered on social exchange rather than on any expectation of direct personal reward. ■ There is broad agreement that consent is not a panacea. Although potential donors should always be asked for their permission in a manner that is sensitive to specific vulnerabilities and desires, it is also generally agreed that people should not be forced to absorb large amounts of technical information or to give recurrent, project-specific consent unless they wish to do so. ■ There is also general agreement that rigorous oversight of biobanks is crucial and that this should combine public control with oversight by institutional ethical and scientific review committees. ■ There is general agreement that it is important to take seriously the results of research, which have consistently revealed high levels of trust, a desire for or expectation of reciprocity, and an expectation of public involvement and benefit sharing.	“Qualitative research shows that donation to biobanks is a complex process shaped by donors’ embeddedness in a number of social contexts; by complex relations of trust in biomedicine; and by the ambiguous status of human tissue. While these findings are theoretically and practically useful, current sociological theorising is very general. A more detailed and nuanced ‘sociology of biobanking’ is needed, and this might be best achieved by exploring specific theoretical questions in a variety of biobanking settings.” (p. 792)
Chan et al. 2012 (24)	Systematic review of 18 qualitative and mixed-methods studies of patient perceptions of research use of residual clinical samples, 1990–2010 (international)	■ All of the authors of the reviewed studies failed to clearly describe the methodology and paradigm used in their research. Thus, the reviewers were unable to assess the congruity (or otherwise) between the stated philosophical perspectives and the research methodology and objectives. ■ Patient consent to the use of leftover tissue is a complex interaction between many factors and not driven solely by perceptions of benefits to self or others. ■ Health care institutions and regulatory authorities must provide clear and transparent safeguards and controls and communicate these to patients prior to the consenting process. ■ Views on ownership and rights to the future use of leftover tissue vary among patients and influence their willingness to consent to further use. ■ Patients have divided views on the use of their leftover tissue for commercial purposes.	“For leftover tissues to be used, patients must clearly understand: the type of consent they are providing (opt in or opt out); the parameters for the future research use of their leftover tissues; the safeguards put into place to protect the individual and the donated tissue from unethical use; and the commercial implications of their consent.” (p. 9)
Rachul et al. 2012 (113)	Systematic review of 87 quantitative and qualitative studies of public perceptions of biobanking, 1996–2011 (international)	■ The public seems relatively comfortable with a variety of consent scenarios; however, when forced to choose a preference, there is little consensus that any particular type of consent would be best. ■ The majority of participants in most studies report being willing to participate in biobank research. ■ There is substantial variation in individual concerns about privacy and who participants think should be able to access their samples. ■ There is some consensus that, when asked hypothetically and generally, the majority of participants report a desire for research results and incidental findings. ■ The ability to withdraw, as a basic human right and/or as a factor in participation decisions, appears to be an area of agreement.	“With few exceptions (e.g., return of results and withdrawal), what is disclosed by this review of survey work is a lack of consensus on key issues, especially in the context of the nature of consent required.” (p. 1)
Nobile et al. 2013 (101)	Systematic review of 13 quantitative and qualitative studies of biobank participants’ reasons for enrolling, 2006–2012 (international)	■ Reasons for enrolling in population biobank studies stem from personal attitudes (altruism, trust, or optimism), subjective perceptions of costs (ease of procedure or an institution’s reputation), subjective perceptions of benefits (personal benefit or benefit to others), and contextual level (family history of disease or pressure from institution/study personnel).	“Our review showed that, next to personal attitudes such as altruism and subjective perception of a participation entailing few burdens and low risk, personal benefit through health-related information is frequently expressed as a motivator to enroll in biobank research among randomly selected participants. Given the fact that our review addressed apparently healthy donors, the magnitude of this expectation is striking and quite unsettling.” (p. 44)
D’Abramo et al. 2015 (31)	Content analysis of 10 quantitative and qualitative studies of biobank participants’ perceptions and views of consent, 2005–2014 (international)	■ The majority of research participants opted for some version of limited consent when being informed about such a possibility. Among the factors influencing the type of preferred consent were information about sponsorship of biobank research by the pharmaceutical industry and participants’ trade-off between privacy and perceived utility. ■ Studies investigating research participants’ understanding and recall regarding the consent procedure indicated considerable lack of both the above aspects. ■ Research participants’ perceptions of benefits and harms differ across those studies.	“Our review suggests that there are two important issues related to how ethically acceptable consent can be elicited in research practice beyond the focus of the theoretical literature on justifications of broader or narrower approaches to consent. Firstly, the choices provided as part of the consent procedure, and secondly, the way in which potential research participants are informed about biobank research.” (p. 8)
Garrison et al. 2016 (43)	Systematic review of 48 quantitative and qualitative studies of public opinion on broad consent and data sharing, 2001–2015 (United States)	■ Although the majority of respondents often expressed support for broad consent when that was the only choice offered, only a minority favored broad consent when other options, such as tiered or study-specific consent, were offered. ■ Willingness to give broad consent increased when data were deidentified, the logistics of biobanks were communicated, and privacy was addressed. ■ Willingness for data to be shared was generally high, but it was lower among individuals from underrepresented minorities, among individuals with privacy and confidentiality concerns, and when pharmaceutical companies had access to data. ■ Although a few studies generally found that men were more likely to support broad consent, most investigators did not examine the impact of gender on attitudes. Although data about race/ethnicity are incomplete, it seems that minorities often have more concerns about broad consent, although existing evidence suggests that these concerns can be ameliorated in some cases by discussion and education. Much less is known about the impact of sociodemographic factors.	“Additional research is needed to understand factors affecting willingness to give broad consent for biobank research and data sharing in order to address concerns to enhance acceptability.” (p. 663)

**Table 3:** Knoppers & Chadwick’s (80) emerging ethical trends in human genetic research

Principle	Description
Reciprocity	The contribution of the research participant, the notion of exchange
Mutuality	The familial nature of genetic information
Solidarity	Common interests and moral responsibilities to each other
Citizenry	The need for public consultation and debate, notions of collective identity
Universality	The common heritage of humanity, that the human genome is shared by all

**Table 4:** Grady et al.’s (47) considerations for obtaining consent for biospecimen research

Benefits	Costs
▪ Shows respect for people ▪ Allows people to control whether their samples are used for research purposes ▪ Allows people to decide whether the risks and burdens of research are acceptable ▪ Allows people to decide whether to contribute to the goals of research, thus protecting and possibly promoting their fundamental values and nonwelfare interests ▪ Increases transparency, thus promoting public trust and the ongoing viability of biospecimen research	▪ Places a burden on donors’ and investigators’ time ▪ Requires resources to obtain consent ▪ Incurs considerable costs and burdens related to maintaining systems that record and honor individual choices or related to later seeking reconsent ▪ Raises the possibility that donors may decline, possibly diminishing the potential for future research

## ASKING PATIENTS ABOUT ACCEPTABLE POLICY OPTIONS

At Duke University’s Program for Empirical Bioethics, we are conducting NIH-funded research (R01-LM-012178) on patient perspectives on research use of electronic health records (EHRs). Through extensive pretesting, we developed educational materials and an interview guide with the goal of obtaining policy-relevant input on topics with which we expected few would be familiar. In the interview, we ask about general notification, broad permission, and categorical consent. For each approach, we provide a verbal description and visual example. We then ask the following questions to confirm that interviewees understand the approach, encourage reflection, and obtain their opinion about its acceptability:

■ Can you please tell me in your own words how [approach] works?
■ What advantages can you think of about using [approach] to let patients know about research using EHRs?
  What disadvantages can you think of?
■ If the place you get your health care used [approach] as its approach, would that be okay?

We discuss these questions for each approach, and then—rather than asking about personal preference—we ask interviewees to weigh in on the policy question:

■ Considering the advantages and disadvantages of all the approaches we discussed, which do you think would be the most appropriate way to let patients know their EHR might be used for research?
